# Educational Disparities in Age-Related Hearing Loss and Hearing Aid Use Across Age, Gender, and European Region

**DOI:** 10.1093/geronb/gbae202

**Published:** 2025-01-29

**Authors:** Donata Stonkute, Yana Vierboom

**Affiliations:** Max Planck Institute for Demographic Research, Rostock, Germany; Helsinki Institute for Demography and Population Health, University of Helsinki, Helsinki, Finland; Max Planck Institute for Demographic Research, Rostock, Germany; Office of Population Research, Princeton University, Princeton, New Jersey, USA; (Social Sciences Section)

**Keywords:** Aging, Education, Health inequalities, Relative Index of Inequalities, SHARE

## Abstract

**Objectives:**

Affecting 1 in 5 adults in Europe, hearing loss (HL) is linked to adverse health outcomes, including dementia. We aim to investigate educational inequalities in hearing health in Europe and how these inequalities change with age, gender, and region.

**Methods:**

Utilizing 2004–2020 data from the Harmonized Survey of Health, Ageing, and Retirement in Europe (SHARE), a representative sample of Europeans aged 50 and older, we analyze: (1) age-standardized prevalence of HL and hearing aid (HA) use among eligible individuals and (2) educational inequalities therein using the Relative Index of Inequality across age, gender, and European regions.

**Results:**

The prevalence of self-reported HL increases with age, is greater among men, and is consistently higher among those with lower levels of education. At age 50–64, particularly in Southern and Eastern Europe, low-educated women experience more than 3 times the risk of HL compared to highly educated women. These inequalities diminish as women age. Northern Europe is a front-runner in meeting HA needs. Southern and Eastern Europe lag behind, with less than 2 in 10 individuals eligible for HAs utilizing them.

**Discussion:**

Substantial variations in the educational gradient of hearing health across age, gender, and European regions underscore the importance of targeting specific subpopulations in efforts to mitigate health inequalities. Of particular concern is the regional discrepancy between the prevalence of HL and the use of HAs. The example of Northern Europe suggests that there is unused potential to improve healthy aging in Europe through enhanced access to HAs.

Age-related hearing loss (ARHL) is among the most prevalent chronic conditions, and a leading cause of disability (Haile et al., 2021). Currently, one in five adults in Europe experiences hearing loss (HL; Eurostat, 2022), a proportion that will increase as the population ages (Jiang et al., 2023).

Educational attainment plays a crucial role in understanding and addressing ARHL. As a fundamental cause of health disparities (Phelan & Link, 2013), education is not only associated with the risk of developing health impairments but also influences the likelihood of seeking and adhering to interventions. Lower levels of education are linked to a higher prevalence of HL (Agrawal et al., 2008; Cruickshanks et al., 2015; Helvik et al., 2009) and a lower likelihood of hearing aid (HA) acquisition and consistent use (Benova et al., 2015; Fischer et al., 2011; Gahlon et al., 2024).

Research indicates that individuals with lower levels of education may be more exposed and susceptible to health risks across contexts (Beckfield et al., 2015) and sociodemographic groups (Diderichsen et al., 2019). Consequently, the educational returns to health are highly heterogeneous even among individuals with seemingly identical educational attainment (Kemp & Montez, 2020). This may also tie into patterns of help-seeking behaviors (Plug et al., 2012). The varied impact of education on health highlights the need to identify vulnerable groups at risk of HL.

Despite this need, studies examining the educational gradient in ARHL and how it varies across contexts are scarce. Addressing this significant gap, we aim to assess the extent of educational disparities in HL and HA use in Europe and how it varies across age, gender, and European region. Our findings will hopefully inform efforts to decrease educational disparities in ARHL, thus reducing inequities in downstream health conditions associated with impaired hearing.

## Background

Age-related hearing loss—also known as presbycusis—results from the gradual deterioration of ear structures, influenced by a complex interplay of factors accumulating throughout an individual’s life (Yamasoba et al., 2013). Environmental factors play a significant role, particularly occupational exposure to noise and ototoxic chemicals. A study found that individuals in manual occupations in England are almost twice as likely to develop HL compared to those in other occupations (Tsimpida et al., 2019), likely due to exposure to noisy environments and ototoxic chemicals (Lewkowski et al., 2019). Lifestyle factors, including smoking (Nomura et al., 2005) and other behaviors associated with cardiovascular disease (Baiduc et al., 2023), further contribute to the development of hearing impairment. Additional factors such as head and ear traumas, nutritional deficiencies, chronic conditions and the use of ototoxic medications also compound over time (World Health Organization, 2021). At the same time, intrinsic biological factors, such as genetic predisposition, hormonal influences, and variations in inner ear structures (Lien & Yang, 2021; Nolan, 2020; Van Eyken et al., 2007), also contribute to the etiology of ARHL.

ARHL, if left unaddressed, can adversely affect various aspects of an older individual’s life, leading to social isolation (Shukla et al., 2020) and depression (Lawrence et al., 2020). Most critically, ARHL has emerged as a significant geriatric health concern due to its established links with cognitive decline and dementia. Research indicates that individuals with impaired hearing face nearly twice the risk of developing dementia compared to those with normal hearing (Livingston et al., 2017).

Fortunately, a range of effective interventions is available for individuals seeking to treat HL. The use of hearing aids appears to mitigate the increased risk of dementia (Yeo et al., 2023), as well as other adverse effects associated with ARHL (Chisolm et al., 2007). Recent evidence from a randomized control trial shows that they significantly reduce the risk of cognitive decline in people already at high risk (Lin et al., 2023), possibly in part through changes in brain structures (de Boer et al., 2022). Despite these benefits, HA uptake remains low. In Western European countries, only 35% of eligible individuals use HAs (Bisgaard & Ruf, 2017).

Both the prevalence of ARHL and the uptake of HAs are subject to social patterning, though current evidence on educational disparities is limited in geographical scope and comparability due to varying definitions of HL (Roth et al., 2011). A study in the United States suggests a clear negative relationship between educational attainment and the risk of HL (Agrawal et al., 2008). Likewise, in the Netherlands, lower education levels are linked to an increased risk of ARHL, even when accounting for other risk factors such as occupational noise exposure (Rigters et al., 2016). However, research in Norway found an educational gradient only for lower-educated men (Helvik et al., 2009).

The impact of education extends to the use of HAs, as it can equip individuals with the necessary knowledge and resources to seek and afford treatment, manage hearing devices, and cope with the societal stigma surrounding HL (Jorbonyan et al., 2021; Weycker et al., 2021). Indeed, in both the United States (Weycker et al., 2021) and Norway (Helvik et al., 2016), those with higher education levels are more likely to consistently use HAs. Taken together, these findings suggest that the groups that could benefit most from HAs are the least likely to use them.

The unequal distribution of the risk factors described above across educational levels may partly explain disparities in hearing health. Individuals with lower levels of education are more likely to engage in deleterious health behaviors such as smoking (Hiscock et al., 2012), have a higher risk of microvascular health conditions (Yusuf et al., 2020), have a higher risk of exposure to harmful occupational noise (Lie et al., 2016), and are less likely to engage in healthcare-seeking behaviors (Lee et al., 2021).

Hearing health additionally varies across age, region, and sex/gender. ARHL naturally increases with age, but its onset and progression are associated with geographic location (Jiang et al., 2023). Sex/gender differences in hearing health are also well-documented, with men generally experiencing greater high-frequency HL due to occupational noise exposure, whereas women may be at higher risk for certain types of hearing disorders related to hormonal changes (Lien & Yang, 2021; Nolan, 2020). Additionally, research from the United States shows that women are more likely to seek help earlier after noticing HL (Humes et al., 2009) and to start using HAs sooner than men (Simpson et al., 2019).

Existing research adds an additional layer of complexity: not only does hearing health vary by subgroup, but the extent of educational disparities in hearing health also differs among subgroups. First, educational returns to health can differ for men and women due to both biological and socially patterned exposures and behaviors (Ross & Mirowsky, 2010). Although estrogen lowers the risk of HL for women relative to men (Shuster et al., 2019), its protective effects are reduced following menopause (Nolan, 2020). Because menopause may commence at an earlier age among women with lower levels of education (Schoenaker et al., 2014), hormonal protective effects may be less pronounced among low-educated women compared to those with higher levels of education. On the other hand, low-educated men are more likely than women to work in occupations that expose them to harmful noise (Lien & Yang, 2021), resulting in a more pronounced gradient in men.

Second, the relationship between education and health is not static across the life course. Some studies suggest that educational disparities in health may widen with age due to cumulative advantage processes (Geronimus et al., 2010), whereas others point to potential convergence in later life (House et al., 1994).

Finally, educational gradients in ARHL likely vary across geographical contexts. Given that comprehensive public health and prevention programs can facilitate hearing health maintenance among older adults (Maharani et al., 2021), variations in access to and quality of hearing care across Europe have been raised as a concern (EFHOH, 2018). In addition to variation in healthcare systems, geographic regions also represent unique epidemiological profiles. For instance, elevated mortality rates from cardiovascular diseases among less-educated males in Eastern Europe (Di Girolamo et al., 2020) may result in a survival bias, with older men representing a more health-selected group, creating an intersection between region, age, and gender. Therefore, a comprehensive understanding of educational disparities in ARHL requires careful consideration of these factors.

## Research Aims

Despite the importance of hearing health in aging populations, comparative European studies on HL and HA usage are scarce, particularly those examining educational disparities across age, gender, and regional dimensions. This knowledge gap hinders the identification of vulnerable populations and impedes evidence-based policymaking for resource allocation in hearing healthcare. Our study addresses this research gap with two primary objectives. First, we aim to estimate both the education-specific prevalence of self-reported HL and HA use and the magnitude of educational inequality therein. Second, we aim to compare how results vary across age, gender, and European region.

## Data and Methods

### Population

We use data from the Harmonized Survey of Health, Ageing, and Retirement in Europe (SHARE) developed by the Gateway to Global Aging Data (https://g2aging.org/). SHARE is conducted biennially since 2004 and is representative of the noninstitutionalized population in Europe aged 50+. It collects information on multiple aspects of individuals’ lives across demographic, socioeconomic, and health sectors. The survey uses a probability-based sampling approach, drawing participants from official population registers whenever possible. In cases where these registers are unavailable, alternative sampling methods are employed. Regular refreshment samples are drawn to maintain representation of younger age cohorts and to offset panel attrition. However, the specifics of the sample design, especially the sampling frame, differ across countries (Bergmann et al., 2017). More details of the design and survey methods are described elsewhere (Börsch-Supan, 2022; Börsch-Supan et al., 2013).

This study is based on a pooled cross-sectional sample from Waves 1 (2004–2005) through 8 (2019–2020), excluding Waves 3 and 7 because they focus on retrospective life histories. We also exclude observations for individuals missing age or younger than 50 years of age (*n* = 5,933), who usually are spouses of the targeted householder. Observations with missing data on outcome and sociodemographic variables (*n* = 1,021) or missing cross-sectional weights (*n* = 758) are additionally excluded, leading to a study population of 288,266 person-wave observations. While SHARE is a longitudinal study, our analysis treats each wave as a separate cross-sectional population.

### Outcome Variables

We use self-reported hearing quality (SRHQ) to assess HL, as it captures subjective experiences beyond audiometric measures. SRHQ better reflects quality of life impacts and treatment-seeking behavior (Manchaiah & Stephens, 2013), providing a holistic measure of hearing disability that accounts for personal, environmental, and social factors influencing HL’s impact. Respondents were asked: “Is your hearing [using a hearing aid as usual]: 1. Excellent; 2. Very good; 3. Good; 4. Fair; 5. Poor?” Consistent with previous research (Hansen et al., 2023; Maharani et al., 2021), we consider any levels less than good as a self-reported HL.

We obtain HA use from the binary question “Are you usually wearing a hearing aid?” We consider HA use only among eligible individuals to facilitate comparison between sociodemographic groups, regardless of differences in baseline hearing levels. We define this groups as: (1) individuals who either report using a HA, regardless of their SRHQ or (2) all respondents who report less than good hearing, regardless of HA use.

### Sociodemographic Variables

The focal stratifying sociodemographic variable we consider is education. We categorize educational levels based on the International Standard Classification of Education (Statistics UIS, 2012): low education (lower secondary education or less, ISCED 0-2); medium education (upper secondary or vocational training, ISCED 3-4); and high education (tertiary education or higher, ISCED 5-6).

We also consider age, gender, and European regions as variables intersecting with education. Following Ferrara’s classification of European welfare regimes (Ferrera, 1996), we classify the 28 European countries in SHARE into four regions: Eastern Europe, Northern Europe, Southern Europe, and Western Europe. Supplementary Table S1 lists the countries in each region.

### Methodological Approach

Our analytical strategy follows three steps. First, we use cross-sectional weights to estimate the prevalence of HL and HA use across 5-year age intervals, by education, gender, and region. We then use the Standard European Population of 2013 to standardize these estimates. Finally, we estimate the Relative Index of Inequality (RII).

SHARE’s cross-sectional respondent weights are calibrated based on the population size and eight gender-age groups to ensure estimates are nationally representative. Although our prevalence estimates across 5-year age groups by gender and education provide a comprehensive view of age-related trends, we face sample size limitations when additionally stratifying by region. To address this, we calculate the prevalence and RIIs using age-standardized rates, thus accounting for differences in age structures across gender, education, and regional groupings.

We complement the age-standardized HL results with estimates stratified by two age groups: 50–64 and 65+. We justify this grouping based on the significant changes typically occurring around age 65, namely retirement, which alter social interactions, finances, and health insurance (Comi et al., 2022; Ebbinghaus, 2021).

The RII, our chosen measure of inequality, considers both differences in population size and the distribution of education across the population (Mackenbach & Kunst, 1997). To calculate the RII, we first rescale the education variable so that the ranked low, medium, and high education values range from 0 to 1, reflecting the midpoints of the cumulative proportion of educational attainment. For example, in the case where a country exhibits an educational distribution of 20% low, 50% medium, and 30% high, the rescaled education values are 0.1, 0.45, and 0.85, respectively.

To obtain the RII, we regress the age-standardized prevalence of HL and HA use against the rescaled education variable. The RII is then expressed by the ratio between predicted outcomes for the lowest and highest levels of education, reflecting the relative disadvantage of belonging to the 0th versus 100th percentile of educational attainment.

We use bootstrap resampling (1,000 samples) to estimate 95% confidence intervals. Although we use percentile intervals (2.5 and 97.5) for most estimates, we use bias-corrected and accelerated intervals for the RII confidence intervals to avoid unrealistic negative RII values due to the nature of linear regression and account for potential skewness in the bootstrap distribution.

All analyses are conducted using R language (version 4.1.1). All replication materials are available at https://github.com/DonataStonkute/hearing_SHARE.

### Supplementary Analyses

We conduct three supplementary analyses to test the robustness of our findings. First, we examine potential underreporting of hearing difficulties by recoding HA users who report good or excellent hearing as having HL. Second, we calculate the Slope Index of Inequality (SII) for educational differences in HL and HA use across regions and genders. Lastly, we limit the open-ended age range when examining the RII from 85+ to 65–79.

## Results

### Sample Characteristics

Table 1 presents the sample characteristics by gender and region, based on 288,266 person-wave observations. The distribution of education varies considerably across regions. Notably, across all regions, women are more likely than men to report lower levels of education.

**Table 1.** Sociodemographic and Hearing Health Profile of the Analytical Sample

Variable	Men (*n* = 127,676)				Women (*n* = 160,590)			
	Northern Europe (*n* = 16,550)	Western Europe (*n* = 52,292)	Southern Europe (*n* = 27,791)	Eastern Europe (*n* = 31,043)	Northern Europe (*n* = 19,139)	Western Europe (*n* = 63,953)	Southern Europe (*n* = 33,891)	Eastern Europe (*n* = 43,607)
Age, mean (*SD*)	67.3 (9.8)	66.3 (9.8)	67.7 (9.9)	66.9 (9.4)	67.1 (10.2)	66.4 (10.4)	67.3 (10.6)	67.4 (9.9)
Age categories, *n* (%)								
50–64	6,943 (42.0)	24,678 (47.2)	11,448 (41.2)	13,729 (44.2)	8,391 (43.8)	30,708 (48.0)	14,945 (44.1)	18,725 (42.9)
65+	9,607 (58.0)	27,614 (52.8)	16,343 (58.8)	17,314 (55.8)	10,748 (56.2)	33,245 (52.0)	18,946 (55.9)	24,882 (57.1)
Education, *n* (%)								
Less than upper secondary	4,921 (29.7)	14,419 (27.6)	18,950 (68.2)	10,101 (32.5)	6,221 (32.5)	25,492 (39.9)	25,292 (74.6)	16,733 (38.4)
Upper secondary and vocational	6,346 (38.3)	22,392 (42.8)	5,291 (19.0)	15,385 (49.6)	5,999 (31.3)	24,749 (38.7)	5,655 (16.7)	20,035 (45.9)
Tertiary	5,283 (31.9)	15,481 (29.6)	3,550 (12.8)	5,557 (17.9)	6,919 (36.2)	13,712 (21.4)	2,944 (8.7)	6,839 (15.7)
Self-rated hearing,^a^ mean (*SD*)	2.6 (1.1)	2.8 (1.0)	2.8 (1.0)	2.9 (1.1)	2.3 (1.0)	2.6 (1.0)	2.7 (1.0)	2.7 (1.0)
Hearing loss,^b^ %	19.8	22.7	22.8	25.2	12.6	15.9	19.1	20.4
Wears a hearing aid, %	14.3	8.9	3.4	4.5	9.4	6.2	2.9	3.2

*Note*s: *SD* = standard deviation. Estimated using harmonized SHARE data, Waves 1–8, excluding 3 and 7.

^a^1 = excellent; 5 = poor.

^b^Self-rated hearing less than good.

The table also provides a summary on SRHQ, where higher values indicate poorer hearing. Women consistently report slightly better SRHQ than men across all regions. Regionally, Eastern Europeans indicate poorer SRHQ, whereas Northern Europeans rate their hearing quality more favorably. These gender and regional patterns are similarly reflected in the crude prevalence of HL. Furthermore, regions experiencing higher crude prevalences of HL tend to show lower rates of HA usage.

The analysis on HA use is restricted to individuals who reported either using an HA or less than good hearing. Consequently, the analytical sample for HA use differs slightly from that presented in Table 1. The sample is characterized by a higher mean age and a larger proportion of individuals with lower levels of education. Further details can be found in Supplementary Table S2.

### Age Trends in Hearing Loss and Hearing Aid Use

**Figure 1.**
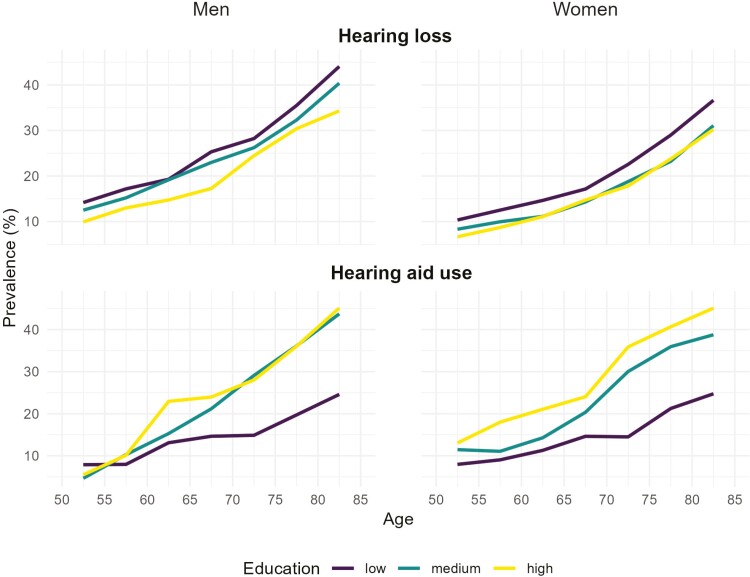
Hearing loss and hearing aid use across age by education and gender. Hearing loss is measured in the total population, whereas hearing aid use is measured among those eligible for HAs. The latter group is defined as individuals who either report using HAs or rate their hearing as less than good. HA = heading aid.

Figure 1 shows age trends in the prevalence of HL across gender and educational levels. There is a consistent increase in HL with age for both men and women, with prevalence rising from around 10% at age 50–55 to over 45% by age 85+ for low-educated men. Gender differences are evident, with men having higher prevalence of HL than women at all ages and levels of education. For example, at age 75–80, highly educated men have a prevalence of HL of 30%, compared to 24% for women with the same level of education. Education also plays a significant role, with lower levels of education associated with higher prevalences of HL.

Patterns of HA use exhibit similar age-related increases as those seen for HL, though with notable differences by gender and educational level. Gender differences in HA use are less pronounced than those for HL, with women showing slightly higher prevalence of HA use in certain age and education categories. Educational level seems to have a significant impact on HA use, particularly among women, where a clear gradient is observed: higher education is consistently associated with greater HA use. Among men, the disparity is most evident between low and medium/high education levels, with medium and high education yielding similar prevalence rates. For instance, by age 80–85, 45% of highly educated men eligible for HAs report using them, compared to only 25% of men with low educational attainment.

### Educational Disparities in Hearing Loss

Table 2 presents education-specific prevalence estimates and relative inequalities in age-standardized HL prevalence among individuals aged 50+, segmented into two age groups (50–64 and 65+). Individuals with lower educational attainment consistently demonstrate a higher prevalence of HL compared to those with higher levels of education. This educational gap is evident across all groups, highlighting the robust association between lower education and increased HL risk. RII estimates further reinforce this positive gradient, ranging from 1.2 to 1.8 for men and 1.3 to 1.8 for women. An RII of 1.0 indicates no inequality in HL prevalence across educational groups, meaning all educational levels have the same risk of HL. RII values above 1.0 show that lower educational attainment is linked to a higher HL risk. For example, men with lower educational attainment are up to 80% more likely to experience HL, whereas women with lower educational attainment face a 30%–80% higher risk of HL compared to their highly educated counterparts. This demonstrates clear educational disparities in HL, though the extent varies substantially by age, gender, and regional context.

**Table 2.** Prevalence of Hearing Loss by Gender, Age, Region, and Education, With Associated Relative Index of Inequality

Variable	Men			Women		
	50–64	65+	Total	50–64	65+	Total
Northern Europe						
ASP by education						
Low	13.5 (11.1–16.1)	25.2 (23.6–26.9)	19.4 (18.0–20.9)	9.1 (7.4–11.0)	17.7 (16.4–18.9)	13.4 (12.3–14.4)
Medium	14.3 (12.5–16.3)	24.4 (22.7–26.2)	19.4 (18.1–20.7)	7.7 (6.5–9.1)	17.2 (15.7–18.8)	12.5 (11.5–13.6)
High	12.3 (10.3–14.3)	21.5 (19.7–23.2)	16.9 (15.5–18.2)	6.5 (5.5–7.5)	15.4 (13.5–17.2)	10.9 (9.9–12.1)
RII	1.1 (0.9–1.9)	1.3 (1.1–1.7)	1.2 (1.1–1.5)	1.7 (1.3–4.0)	1.2 (1.0–1.7)	1.4 (1.2–1.9)
Western Europe						
ASP by education						
Low	18.8 (16.8–20.9)	35.6 (34.2–37.0)	27.2 (26.0–28.6)	13.1 (11.3–15.2)	25.2 (24.2–26.3)	19.2 (18.1–20.4)
Medium	17.9 (16.5–19.3)	31.4 (30.0–32.9)	24.7 (23.6–25.7)	11.4 (10.4–12.4)	23.4 (22.0–24.8)	17.4 (16.5–18.2)
High	12.5 (11.2–14.1)	28.4 (26.8–30.1)	20.5 (19.4–21.6)	10.1 (9.0–11.3)	22.0 (20.2–23.8)	16.1 (14.9–17.3)
RII	1.7 (1.5–2.6)	1.4 (1.3–1.6)	1.5 (1.4–1.7)	1.4 (1.2–2.2)	1.2 (1.1–1.5)	1.3 (1.2–1.6)
Southern Europe						
ASP by education						
Low	16.0 (14.1–18.0)	34.3 (33.3–35.5)	25.1 (24.1–26.3)	12.3 (10.7–14.2)	29.7 (28.8–30.7)	21.0 (20.1–22.1)
Medium	10.1 (8.2–12.2)	27.6 (24.4–30.8)	18.9 (17.1–20.8)	5.4 (4.4–6.6)	21.2 (18.1–24.5)	13.3 (11.7–15.1)
High	12.6 (7.6–18.4)	22.2 (18.9–25.4)	17.4 (14.1–20.7)	6.6 (4.4–9.3)	27.5 (22.6–32.3)	17.1 (14.5–19.9)
RII	1.6 (1.0–4.3)	1.9 (1.6–2.4)	1.8 (1.5–2.4)	3.3 (2.2–8.8)	1.4 (1.2–1.9)	1.6 (1.4–2.3)
Eastern Europe						
ASP by education						
Low	16.2 (12.9–19.1)	37.2 (35.1–39.8)	26.7 (24.8–28.5)	11.6 (9.9–13.5)	31.7 (30.1–33.6)	21.7 (20.5–23.0)
Medium	13.5 (11.8–15.1)	31.6 (29.2–33.8)	22.5 (21.1–24.0)	8.9 (7.7–10.1)	25.6 (23.1–28.2)	17.2 (15.8–18.7)
High	11.0 (8.5–13.3)	28.4 (24.8–31.9)	19.7 (17.7–22.0)	4.4 (3.0–6.1)	23.2 (19.9–26.4)	13.8 (12.0–15.6)
RII	1.6 (1.2–3.1)	1.5 (1.3–1.9)	1.5 (1.3–1.9)	3.3 (2.3–8.5)	1.6 (1.4–2.1)	1.8 (1.6–2.3)

*Notes*: ASP = age-standardized prevalence; RII = Relative Index of Inequality. Values represent prevalence of hearing loss and Relative Index of Inequalities estimates (95% confidence intervals).

### Heterogeneity Across Age, Gender, and Region

Age-related patterns emerge as a significant factor in the heterogeneity of educational inequalities in HL. Women experience more pronounced educational inequalities at ages 50–64 than at later ages. Although less-educated women still face an increased risk of HL after age 65, the gap narrows, particularly in Southern and Eastern Europe, where RIIs drop from 3.3 to 1.4 and 1.6, respectively. In contrast, educational inequalities among men either widen or narrow with age depending on the region.

Women generally experience greater educational inequalities in HL prevalence than men at ages 50–64. However, this gender gap appears to diminish or even reverse in the 65+ age group across most regions.

There are considerable and consistent regional variations in educational inequalities in HL. In Southern and Eastern Europe, the educational gradients are larger than in Northern and Western Europe. For example, Eastern European women exhibit an RII of 1.8, indicating nearly twice the relative risk of HL among low-educated women compared to their highly educated counterparts.

Although we generally observe a negative association between educational attainment and HL prevalence, exceptions exist. Southern Europe exhibits a U-shaped pattern, with low- and highly educated individuals, particularly women, reporting higher HL prevalence than those with medium education.

### Hearing Aid Use Among Eligible Individuals

The pattern of HA use among individuals eligible for HAs in Table 3 does not reflect the linear association observed between education and HL prevalence, or the one observed in Figure 1 for all regions combined. The RII for HA use fluctuates above and below 1, indicating no consistent educational gradient. In some instances, particularly among women, education-specific prevalence estimates suggest an inverted U-shaped pattern, with medium education showing higher prevalence than low and high educational attainment.

**Table 3.** Prevalence of Hearing Aid Use Among Eligible Individuals by Gender, Region, and Education, With Associated Relative Index of Inequality

Gender	Region	Education			RII
		Low	Medium	High	
Men	Eastern Europe	10.9 (8.8–13.7)	12.5 (10.2–15.1)	12.9 (9.5–16.4)	0.8 (0.6–1.9)
	Northern Europe	34.1 (30.5–38.0)	37.1 (334.6–39.5)	40.3 (36.6–44.1)	0.8 (0.6–1.1)
	Southern Europe	10.5 (8.9–12.4)	12.3 (9.3–15.8)	9.0 (6.2–12.2)	1.2 (0.8–4.0)
	Western Europe	18.9 (14.8–18.4)	23.1 (18.3–20.8)	25.2 (19.3–22.8)	0.7 (0.6–1.0)
Women	Eastern Europe	14.5 (10.2–19.2)	11.8 (9.5–14.7)	13.1 (7.9–20.4)	1.4 (0.6–4.1)
	Northern Europe	36.6 (32.7–40.5)	42.7 (37.0–48.1)	37.5 (33.8–41.0)	0.9 (0.8–1.3)
	Southern Europe	9.3 (8.2–10.7)	16.6 (12.5–21.7)	12.3 (7.7–17.5)	0.4 (0.1–1.3)
	Western Europe	19.7 (18.2–21.5)	23.4 (21.4–25.4)	29.3 (26.2–32.8)	0.5 (0.4–0.8)

*Notes*: HA = hearing aid; RII = Relative Index of Inequality. The analytic sample for the prevalence of HA use among eligible individuals was restricted to individuals who reported: (1) using an HA or (2) having less than good hearing. This specific analytic sample consists of 68,356 observations. When considering all stratifying variables, cell sizes were too small to further subdivide the analysis to compliment HA use patterns by age subdivision. Values represent prevalence of hearing aid use and Relative Index of Inequalities estimates (95% confidence intervals).

Although there are no discernible patterns in educational inequalities in HA use, regional disparities are particularly pronounced, even more so than in HL prevalence. Consistent with the patterns highlighted in Table 1, regions with lower prevalence of HL exhibit greater HA use among eligible persons. For example, in Southern Europe, less than 1 in 10 men and women with low education eligible for HAs use them, whereas in Northern Europe over 3 in 10 eligible individuals report using HAs. Gender does not appear to play a significant role in shaping either the level of HA use among eligible persons or the extent of educational gradient in HA use.

### Supplementary Analyses

We conduct three supplementary analyses to enhance the robustness of our findings. First, we reestimate educational inequality slopes for HL by redefining the variable to include all HA users, even if they report good or excellent hearing, thereby addressing potential underreporting of hearing difficulties. Although the inequality slopes change only slightly (see Supplementary Figure S1), the HL prevalence levels change substantially, bringing Northern Europe more in line with other regions and showing higher HL rates among men.

Next, we estimate SII to show absolute inequalities in the prevalence of HL and HA use (see Supplementary Table S3). Absolute educational inequalities are consistent with relative inequality measure.

Finally, we restrict the age range to 65–79 years to examine potential age-related convergence in HL prevalence across educational levels (see Supplementary Figure S2). We find larger inequalities among individuals under 80, except in Western Europe and among Eastern European men, suggesting that HL levels might converge across the educational gradient beyond age 80.

## Discussion

This study reveals key findings on educational inequalities in HL and HA use across Europe. We find a consistent negative educational gradient in HL prevalence, with higher education associated with lower HL prevalence. We also find that these inequalities vary significantly by age, gender, and region. Compared to men, women (especially in Southern and Eastern Europe) show more pronounced educational inequalities at ages 50–64. Regional variations are substantial, with Southern and Eastern Europe displaying larger educational gradients than Northern and Western Europe. Although we find no consistent educational gradient in HA use, we observe significant regional disparities in HA use. Those regions with the highest rates of HA usage, such as Northern Europe, are also those with the lowest prevalence of HL.

Our findings largely align with existing literature on hearing health disparities. First, we find a consistent educational gradient in HL across all regions and demographic groups, as previously found in the United States (Agrawal et al., 2008) and the Netherlands (Rigters et al., 2016). Second, men have a higher prevalence of HL across all strata. Previous studies are consistent with this observation, but the magnitude of the gender difference varies according to the definition and typology of HL (Roth et al., 2011). Consistent with Jiang et al. (2023), we find that HL is more prevalent in Eastern and Southern Europe. Contrary to previous studies (Helvik et al., 2016; Weycker et al., 2021), we found no consistent educational gradient in HA use by region and gender. This finding may be attributable to sample size limitations, as pooling data across regions reveals substantial educational differences in HA use (Figure 1).

Our study expands current knowledge by providing a comprehensive comparison of hearing health inequalities across age, gender, and European regions, offering better understanding into how these disparities manifest across diverse contexts. First, findings reveal a complex interplay between age and educational inequalities in HL. We observe what is known as an “age-as-leveler” effect, particularly among women. This effect refers to the phenomenon where health inequalities tend to decrease with age (House et al., 1994). Specifically, we find that educational disparities in HL are more pronounced in the 50–64 age group but narrow significantly in the 65+ group, especially in Southern and Eastern Europe. This challenges the cumulative advantage hypothesis, which suggests health inequalities should widen with age (Geronimus et al., 2010). Indeed, results from our sensitivity analyses, which restricted the 65+ age interval to 65–79 and compared RII estimates with the open-ended age interval, support the convergence of HL levels across the educational gradient beyond age 80.

Several factors could explain the age-leveling effect. Selective mortality may lead to steeper educational gradients in nonlethal conditions for women than for men, as less-educated men are more likely to be affected by lethal conditions, leaving a healthier surviving population (Crimmins et al., 2019).

It is similarly plausible that cohort effects contribute to the observed age patterns as the relationship between education and health may vary throughout the life course (Lynch, 2003). Previous research shows that age-related health inequalities vary by welfare state regime, with stronger associations in Southern Europe (Bambra et al., 2010). Notably, recent cohorts in Southern Europe exhibit reduced health inequalities, likely due to political and social shifts (Navarro et al., 2003). Besides selection and cohort effects, the natural process of aging might overshadow socioeconomic differences in later life, as hearing deterioration becomes more universal (Cruickshanks et al., 2015).

Leveling across age is not consistent across genders, but is stronger among women in all regions, particularly in Southern and Eastern Europe. This pattern suggests that education may be a more powerful stratifier for women’s hearing health in early older adulthood, but its influence wanes in later life. Several factors could explain this gender-specific pattern.

Women in many European countries, particularly in Southern and Eastern regions, have historically faced greater barriers to education, employment, and traditional gender norms, which could exacerbate health inequalities in early older adulthood (Artazcoz et al., 2004). However, as women age, biological factors related to HL, such as decreased levels of estrogen during menopause, might become more dominant (Nolan, 2020), potentially equalizing the impact of earlier social disadvantages. More pronounced selective mortality among men in Eastern Europe, as previously noted, may also be a contributing factor.

Region plays a pivotal role not only in HL prevalence but also in the levels of HA use. We find that HA use among eligible persons in Northern Europe is significantly higher than in other regions, coinciding with a substantially lower HL prevalence and educational disparities therein. Our sensitivity analysis exploring the definition of HL suggests that without high HA use, the prevalence of HL in the Northern Europe would be more in line with that of other regions.

These regional differences provide insights into societal elements such as social safety nets, healthcare accessibility, and levels of health literacy (Beckfield et al., 2015; Sørensen et al., 2015). For example, the pronounced educational disparities in Southern Europe could stem from a healthcare system with limited coverage (Eikemo et al., 2008), whereas larger inequalities in Eastern Europe could reflect historically wide socioeconomic disparities in health behaviors and differential access to quality healthcare (Rechel et al., 2013). Northern Europe, with its relatively low educational inequalities in HL, illustrates that large educational inequalities in hearing health are not inevitable.

A driving force in regional differences is likely the variation in service delivery across European countries, highlighted in a European HA provision report (EFHOH, 2018). These variations encompass differences in financial coverage for HAs, waiting times, consumer choice, and the quality of follow-up care, including counseling. These findings also align with a study by Maharani et al. (2021), which finds that countries with high-performing healthcare systems exhibit reduced levels of HL.

### Strengths and Limitations

We produce representative, age-adjusted prevalence rates and a robust inequality indicator that accounts for regional variations in the educational composition. Self-reported hearing quality captures subjective experiences relating to quality of life better than objective measures, highlighting who may benefit from interventions (Manchaiah & Stephens, 2013). However, educational background may bias reporting, with higher-educated individuals overstating difficulties and lower-educated individuals underreporting them, potentially masking the educational gradient in HL (Choi et al., 2016; Kamil et al., 2015; Tsimpida et al., 2020).

As in most social science, our results may be affected by reverse causation. Although childhood HL can impact educational attainment (Hendry et al., 2021), the vast majority of HL in our sample likely occurred in adulthood, given global age patterns of HL (Haile et al., 2021). This mitigates, but does not eliminate, concerns about reverse causality.

Our pooled data approach provides robust mid-period estimates of hearing health across the study period, but there is temporal heterogeneity in data availability across countries and regions. Many Eastern European countries joined the SHARE project in Wave 4, meaning their data are weighted more toward later periods. However, since educational gradients in health outcomes tend to remain stable over short time frames (Krokstad et al., 2002), slight variations in sample years should not significantly affect our findings.

Additionally, SHARE excludes institutionalized populations and thus may over-represent healthier and more educated individuals, potentially underestimating health inequalities. The varying proportion of institutionalized individuals across countries complicates cross-country comparisons (Permanyer & Spijker 2020). Although we utilize SHARE’s cross-sectional weights to adjust for the age-gender population structure of each country in a given year, these do not fully account for the potential underrepresentation of individuals with lower education or hearing impairments. Our cross-sectional approach mitigates concerns about attrition effects, and existing literature suggests that attrition biases in studying educational health disparities using SHARE data may be limited (Muszyńska-Spielauer & Spielauer, 2022; Spitzer, 2020). If present, any bias would likely result in an *under*estimation of educational inequalities.

## Conclusions

Substantial differences in the educational gradient across age, gender, and regions challenge simplistic, universal models of health inequality, highlighting the importance of sociodemographic and contextual factors. Educational inequalities in HL are most pronounced during midlife but tend to decrease with age, particularly among women. Although education is not a strong predictor of HA use among eligible individuals, contextual factors play a significant role. The role of geographical region on hearing health is unequivocal, with Northern Europe exhibiting a lower prevalence and educational inequalities in HL and leading in HA uptake, whereas Eastern and Southern Europe show the opposite. Collectively, these findings emphasize the need for improved access to HA services in Europe and targeted interventions to promote more equitable hearing health.

## Supplementary Material

gbae202_suppl_Supplementary_Material

## Data Availability

This study uses data or information from the Harmonized Survey of Health, Ageing, and Retirement in Europe data set and Codebook, Version F as of February 2023, developed by the Gateway to Global Aging Data. For more information, please refer to www.g2aging.org. All replication materials are available at https://github.com/DonataStonkute/hearing_SHARE.
