# Supplementary material for: Educational Disparities in Age-Related Hearing Loss and Hearing Aid Use Across Age, Gender, and European Region
Source: J Gerontol B Psychol Sci Soc Sci. 2025 Jan 29;80(3):gbae202. doi: 10.1093/geronb/gbae202 (PMC11822204; doi:10.1093/geronb/gbae202)
Supplement: gbae202_suppl_Supplementary_Material [file gbae202_suppl_supplementary_material.docx]

***The Journals of Gerontology, Series B: Psychological Sciences and Social Sciences* Supplementary Material: Stonkute & Vierboom. Educational Disparities in Age-related Hearing Loss and Hearing Aid Use across Age, Gender, and European Region.**

**Supplementary Table 1.** Classification of Countries by Region

| **Region** | **Country** |
| --- | --- |
| Eastern Europe | Bulgaria |
|  | Croatia |
|  | Czechia |
|  | Estonia |
|  | Hungary |
|  | Latvia |
|  | Lithuania |
|  | Poland |
|  | Romania |
|  | Slovakia |
|  | Slovenia |
| Northern Europe | Denmark |
|  | Finland |
|  | Sweden |
| Southern Europe | Cyprus |
|  | Greece |
|  | Italy |
|  | Malta |
|  | Portugal |
|  | Spain |
| Western Europe | Austria |
|  | Belgium |
|  | France |
|  | Germany |
|  | Ireland |
|  | Luxembourg |
|  | Netherlands |
|  | Switzerland |

**Supplementary Table 2.** Sample Characteristics for Analysis on the Prevalence of HA Use.

| **Variable** | | **Men (N=35 186)** | | | | **Women (N=33 170)** | | | |
| --- | --- | --- | --- | --- | --- | --- | --- | --- | --- |
|  |  | Northern Europe  (N=4 981) | Western Europe (N=14 852) | Southern Europe  (N=6 799) | Central and Eastern Europe  (N=8 554) | Northern Europe  (N=3 726) | Western Europe (N=12 828) | Southern Europe  (N=6 968) | Central and Eastern Europe  (N=9 648) |
| **Age, mean (SD)** | | 71.6 (± 9.9) | 70.6 (± 10.2) | 72.7 (± 10.1) | 71.5 (± 9.7) | 73.4 (± 10.8) | 72.3 (± 11.2) | 74.2 (± 10.9) | 73.3 (± 10.0) |
| **Age categories, n (%)** | |  |  |  |  |  |  |  |  |
|  | 50-64 | 1 262 (25.3) | 4 507 (30.3) | 1 558 (22.9) | 2 272 (26.6) | 860 (23.1) | 3 527 (27.5) | 1 470 (21.1) | 2 064 (21.4) |
|  | 65+ | 3 719 (74.7) | 10 345 (69.7) | 5 241 (77.1) | 6 282 (73.4) | 2 866 (76.9) | 9 301 (72.5) | 5 498 (78.9) | 7 584 (78.4) |
| **Education, n (%)** | |  |  |  |  |  |  |  |  |
|  | Low | 1 693 (34.0) | 4 925 (33.2) | 5 445 (80.1) | 3 351 (39.2) | 1 615 (43.3) | 6 319 (49.3) | 6 092 (87.4) | 4 848 (50.2) |
|  | Medium | 1 882 (37.8) | 6 231 (42.0) | 862 (12.7) | 3 879 (45.3) | 1 127 (30.2) | 4 277 (33.3) | 554 (8.0) | 3 621 (37.5) |
|  | High | 1 406 (28.2) | 3 696 (24.9) | 492 (7.2) | 1 324 (15.5) | 984 (26.4) | 2 232 (17.4) | 322 (4.6) | 1 179 (12.2) |
|  |  |  |  |  |  |  |  |  |  |
| **Self-rated hearing, mean (SD)** | |  |  |  |  |  |  |  |  |
| (1 = excellent; 5 = poor) | | 3.6 (± 0.9) | 3.9 (± 0.8) | 4.10 (± 0.6) | 4.11 (± 0.6) | 3.6 (± 0.9) | 3.8 (± 0.8) | 4.1 (± 0.6) | 4.101 (± 0.6) |
|  |  |  |  |  |  |  |  |  |  |
| **Hearing loss, %** | |  |  |  |  |  |  |  |  |
| (self-rated hearing less than good) | | 65.9 | 79.7 | 93.3 | 91.4 | 64.8 | 79.3 | 93.1 | 92.4 |
|  |  |  |  |  |  |  |  |  |  |
| **Wears a hearing aid, %** | | 47.6 | 31.0 | 13.8 | 16.5 | 48.3 | 31.1 | 14.2 | 14.6 |

**Supplementary Table 3.** Slope Index of Inequality (SII) across Region and Gender.

|  | **Men** | **Women** |
| --- | --- | --- |
|  | **Hearing Loss** | |
| Northern Europe | 3.1 (1.0 – 7.1) | 3.8 (2.1 – 7.0) |
| Western Europe | 8.2 (6.4 – 11.2) | 3.5 (1.9 – 7.3) |
| Southern Europe | 11.6 (8.1 – 17.0) | 7.9 (5.4 – 13.8) |
| Eastern Europe | 7.8 (5.2 – 13.7) | 10.0 (8.0 – 14.0) |
|  | **Hearing Aid Use** | |
| Northern Europe | -8.5 (-14.4 – 3.1) | -2.8 (-8.7 – 11.9) |
| Western Europe | -6.4 (-9.4 – -0.2) | -12.4 (-16.5 – -6.4) |
| Southern Europe | 1.9 (-1.9 –10.4) | -8.5 (-14.3 – 0.7) |
| Eastern Europe | -2.0 (-5.9 – 7.3) | 3.6 (-4.5 – 19.1) |


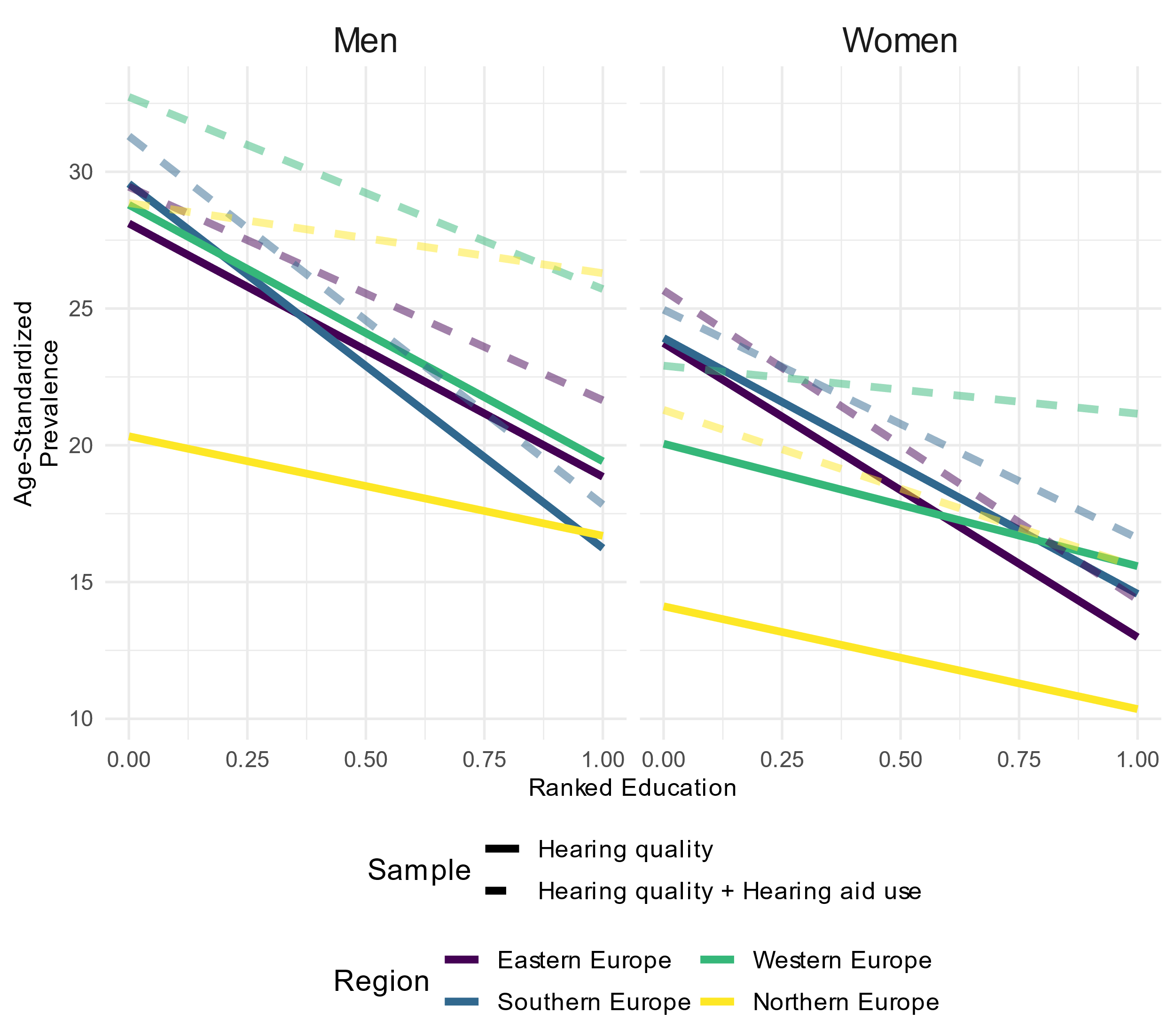


**Supplementary Figure 1.** Comparing Fitted Trends in Educational Inequality: Age-Standardized Prevalence of Hearing Loss by Gender and Region with Original vs. Recoded Hearing Loss Outcome Variables.


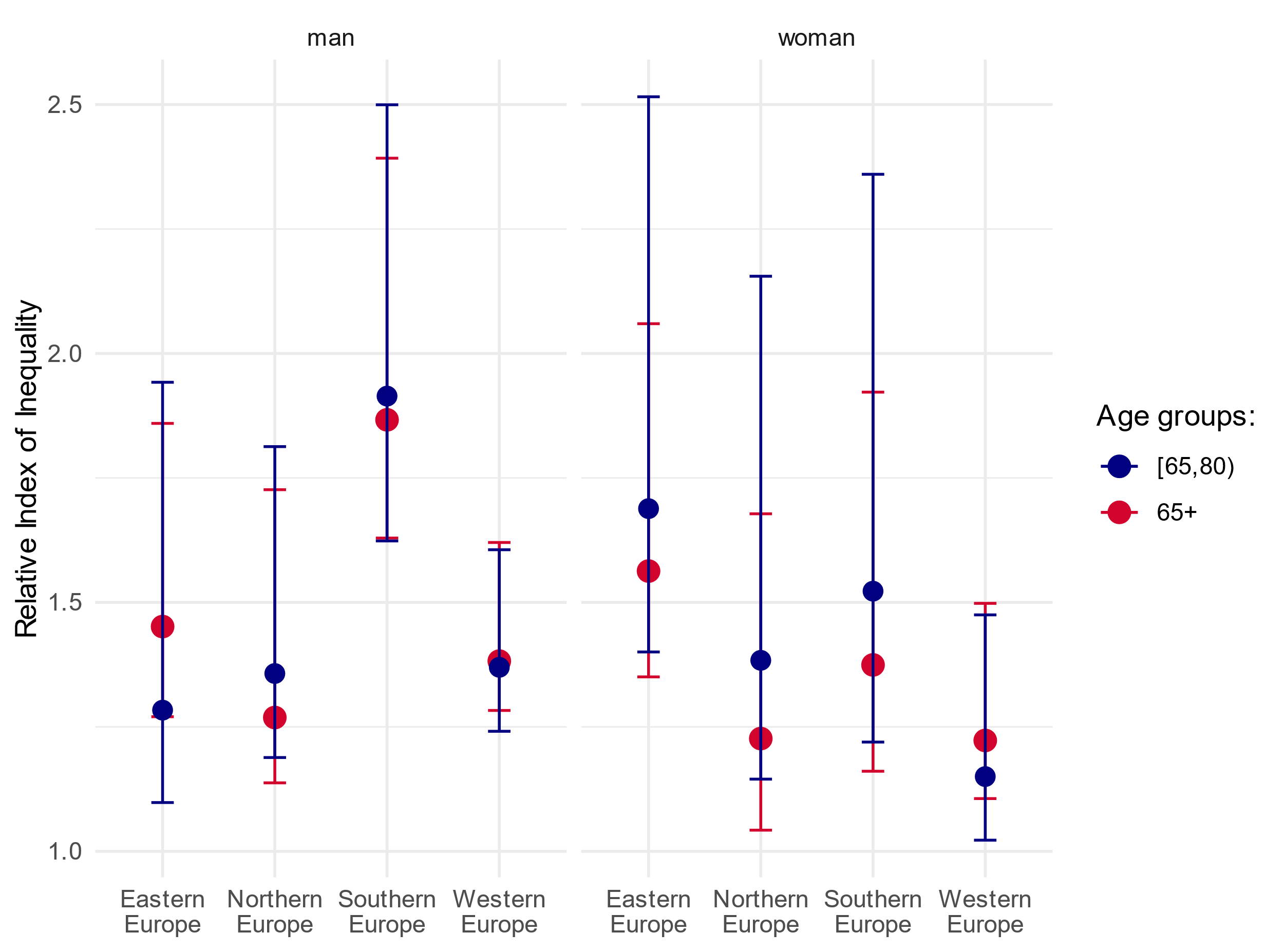


**Supplementary Figure 2.** Age-Restricted Analysis of Hearing Loss Inequalities

**Note.** The analysis was performed using analytical sample of waves 1 through 8, excluding waves 3 and 7.
